# Supplementary material for: Empirical methods for controlling false positives and estimating confidence in ChIP-Seq peaks
Source: BMC Bioinformatics. 2008 Dec 5;9:523. doi: 10.1186/1471-2105-9-523 (PMC2628906; doi:10.1186/1471-2105-9-523)
Supplement: Additional File 1 — A variety of html documents from the USeq web site detailing the available applications, their best usage, output file type descriptions, command line menus, etc. [file 1471-2105-9-523-S1.zip › USeqUserGuides/JavaDoc.zip!/JavaDoc/edu/utah/seq/analysis/ScanSeqs.html]

ScanSeqs


|  |  |  |
| --- | --- | --- |
| **Overview** \| **Package** \| **Class** \| **Use** \| **Tree** \| **Deprecated** \| **Index** \| **Help** | |  |
| **PREV CLASS**   NEXT CLASS | **FRAMES**    **NO FRAMES**     **All Classes** |  |
| SUMMARY: NESTED \| FIELD \| CONSTR \| METHOD | DETAIL: FIELD \| CONSTR \| METHOD |  |


---


## edu.utah.seq.analysis Class ScanSeqs

```
java.lang.Object
  edu.utah.seq.analysis.ScanSeqs
```

---

``` public class ScanSeqs extends java.lang.Object ```

Takes PointData and generates sliding window scan statistics.
Can also use control data to generate a null score distribution and estimate FDRs.

**Author:**
:   nix

---

| **Field Summary** | |
| --- | --- |
| `static int` | `FDR_ID` |
| `static int` | `PVALUE_ID` |
| `static int` | `QVALUE_ID` |
| `static java.lang.String[]` | `SCORE_DESCRIPTIONS` |
| `static int` | `SCORE_ID` |
| `static java.lang.String[]` | `SCORE_NAMES` |
| `static java.lang.String[]` | `SCORE_UNITS` |
| `static int` | `SUMC_ID` |
| `static int` | `SUMT_ID` |


**Constructor Summary**

`ScanSeqs(java.lang.String[] args)`


| **Method Summary** | |
| --- | --- |
| `int` | `calculateNumberControlERs(float threshold)`             Given a score threshold, returns the number of EnrichedRegions that would be generated using the precomputed values. |
| `void` | `calculatePoissonPValues(int maxTotal, SmoothingWindow[] sm, int scoreIndexToConvert, int pValueIndexToPlace, double lambda)`             Makes a call to R to use it to calculate Bonferroni corrected Poisson pvalues. |
| `java.util.HashMap<java.lang.String,PointData>[]` | `fetchControls()`             Fetch control Data for each directory, will merge strands if needed. |
| `static void` | `main(java.lang.String[] args)` |
| `void` | `makeWindows()`             Makes a common set of windows using merged positions. |
| `static float[][]` | `normScrLookUpTable(int n)`             Make a look up table for (t-c)/(Math.sqrt(t+c) returning tc[t][c]. |
| `static void` | `printDocs()` |
| `void` | `processArgs(java.lang.String[] args)`             This method will process each argument and assign new varibles |
| `void` | `savePointAndHeatMapData(java.io.File saveDir, int scoreIndex, int scoreID, SmoothingWindow[] sm, Info info, boolean twoColor)`             Saves point and heat map bar files of a given score. |
| `void` | `saveSmoothedHeatMapData(int scoreIndex, SmoothingWindow[] sm, Info info, java.io.File dir, java.lang.String description, java.lang.String units, boolean twoColor)` |
| `void` | `saveSmoothedPointData(int scoreIndex, SmoothingWindow[] sm, Info info, java.io.File dir, java.lang.String description, java.lang.String units)` |
| `void` | `scorePairedControls()`             Scanner for generating the null control vs control data. |

**Methods inherited from class java.lang.Object**

`equals, getClass, hashCode, notify, notifyAll, toString, wait, wait, wait`

**Field Detail**

### SCORE\_ID

```
public static final int SCORE_ID
```

**See Also:**: Constant Field Values

---


### SUMT\_ID

```
public static final int SUMT_ID
```

**See Also:**: Constant Field Values

---


### SUMC\_ID

```
public static final int SUMC_ID
```

**See Also:**: Constant Field Values

---


### PVALUE\_ID

```
public static final int PVALUE_ID
```

**See Also:**: Constant Field Values

---


### QVALUE\_ID

```
public static final int QVALUE_ID
```

**See Also:**: Constant Field Values

---


### FDR\_ID

```
public static final int FDR_ID
```

**See Also:**: Constant Field Values

---


### SCORE\_NAMES

```
public static final java.lang.String[] SCORE_NAMES
```

---


### SCORE\_DESCRIPTIONS

```
public static final java.lang.String[] SCORE_DESCRIPTIONS
```

---


### SCORE\_UNITS

```
public static final java.lang.String[] SCORE_UNITS
```


**Constructor Detail**

### ScanSeqs

```
public ScanSeqs(java.lang.String[] args)
```


**Method Detail**

### calculatePoissonPValues

```
public void calculatePoissonPValues(int maxTotal,
                                    SmoothingWindow[] sm,
                                    int scoreIndexToConvert,
                                    int pValueIndexToPlace,
                                    double lambda)
```

:   Makes a call to R to use it to calculate Bonferroni corrected Poisson pvalues.
    R has an unusually good estimator that extends very deeply before hitting 0.

---


### normScrLookUpTable

```
public static float[][] normScrLookUpTable(int n)
```

:   Make a look up table for (t-c)/(Math.sqrt(t+c) returning tc[t][c].
    N defines an edge, n\*n the number of cells.

---


### calculateNumberControlERs

```
public int calculateNumberControlERs(float threshold)
```

:   Given a score threshold, returns the number of EnrichedRegions that would be generated using the
    precomputed values. This is a way of minimizing the memory requirements.

---


### makeWindows

```
public void makeWindows()
```

:   Makes a common set of windows using merged positions.

---


### fetchControls

```
public java.util.HashMap<java.lang.String,PointData>[] fetchControls()
```

:   Fetch control Data for each directory, will merge strands if needed.

---


### scorePairedControls

```
public void scorePairedControls()
```

:   Scanner for generating the null control vs control data.

---


### savePointAndHeatMapData

```
public void savePointAndHeatMapData(java.io.File saveDir,
                                    int scoreIndex,
                                    int scoreID,
                                    SmoothingWindow[] sm,
                                    Info info,
                                    boolean twoColor)
```

:   Saves point and heat map bar files of a given score.

---


### saveSmoothedHeatMapData

```
public void saveSmoothedHeatMapData(int scoreIndex,
                                    SmoothingWindow[] sm,
                                    Info info,
                                    java.io.File dir,
                                    java.lang.String description,
                                    java.lang.String units,
                                    boolean twoColor)
```

---


### saveSmoothedPointData

```
public void saveSmoothedPointData(int scoreIndex,
                                  SmoothingWindow[] sm,
                                  Info info,
                                  java.io.File dir,
                                  java.lang.String description,
                                  java.lang.String units)
```

---


### main

```
public static void main(java.lang.String[] args)
```

---


### processArgs

```
public void processArgs(java.lang.String[] args)
```

:   This method will process each argument and assign new varibles

---


### printDocs

```
public static void printDocs()
```


---


|  |  |  |
| --- | --- | --- |
| **Overview** \| **Package** \| **Class** \| **Use** \| **Tree** \| **Deprecated** \| **Index** \| **Help** | |  |
| **PREV CLASS**   NEXT CLASS | **FRAMES**    **NO FRAMES**     **All Classes** |  |
| SUMMARY: NESTED \| FIELD \| CONSTR \| METHOD | DETAIL: FIELD \| CONSTR \| METHOD |  |


---
